# Effect of Icariin on Engineered 3D-Printed Porous Scaffolds for Cartilage Repair

**DOI:** 10.3390/ma11081390

**Published:** 2018-08-09

**Authors:** Ranjith Kumar Kankala, Feng-Jun Lu, Chen-Guang Liu, Shan-Shan Zhang, Ai-Zheng Chen, Shi-Bin Wang

**Affiliations:** 1 Institute of Biomaterials and Tissue Engineering, Huaqiao University, Xiamen 361021, China; ranjithkankala@hqu.edu.cn (R.K.K.); 18749677132@163.com (F.-J.L.); e_luo@sina.cn (C.-G.L.); 1611315042@hqu.edu.cn (S.-S.Z); 2 Fujian Provincial Key Laboratory of Biochemical Technology, Huaqiao University, Xiamen 361021, China

**Keywords:** tissue engineering, 3D printing, icariin, chondrocyte, gelatin, sodium alginate

## Abstract

In recent times, cartilage defects have been the most common athletic injuries, often leading to dreadful consequences such as osteoarthritis, pain, joint deformities, and other symptoms. It is also evident that damage to articular cartilage is often difficult to recover or self-heal because of poor vascular, nervous, and lymphatic supplies. Moreover, cartilage cells have poor regeneration ability and high maturity. Inspired by these facts and the rapid advances in the field of tissue engineering (TE), we fabricated highly porous three-dimensional (3D) scaffold architectures based on cell-responsive polymeric inks, i.e., sodium alginate and gelatin (SA-Gel, 1:3 ratio), by a novel 3D printing method. Moreover, the effect of various processing parameters was systematically investigated. The printed scaffolds of polymer composites gels with excellent transparency, moderate viscosity, and excellent fluid properties showed good surface morphology, better thermal stability and swelling effect, and unique interconnected porous architectures at the optimized operating parameters. In vitro cell proliferation experiments of these cytocompatible scaffolds showed the excellent adhesion rate and growth behavior of chondrocytes. In addition, the porous architectures facilitated the efficient distribution of cells with only a few remaining on the surface, which was confirmed by confocal laser scanning microscopic (CLSM) observations. Icariin (ICA) addition at a concentration of 10 μg/mL further significantly enhanced the proliferation of chondrocytes. We envision that these cell-responsive polymeric inks in the presence of growth regulators like ICA may have potential in engineering complex tissue constructs toward diverse applications in TE.

## 1. Introduction

Recently, tissue engineering (TE) has gained enormous attention due to an increase in the demand for organ replacement therapies and a lack of donated organs [1]. Conceptually, TE is a scientific discipline that integrates the principles of various fields, including but not limited to molecular biology, chemistry, materials science, and engineering, to fabricate and develop biomimetic substitutes while considering various physicochemical as well as biochemical cues for tissue repair and the improvement of tissue biological functions [2,3,4,5,6,7,8,9,10]. Often, the engineering of biomimetic architectures is successful through the generation of highly organized and functional three-dimensional (3D) porous constructs, since the native tissue consists of multiple cell types and various biochemical cues that are responsible for diverse functionalities [1,6]. In addition, the generation of scaffolds with desired interconnected porous structures that are embedded with various biochemical cues often enables better control over the microenvironment and significantly guides the tissue growth along with the recovery of its functionalities [6].

Numerous approaches have been explored to generate 3D porous scaffolds for TE, especially chondrocyte repair such as electrospinning, phase separation, microsphere sintering via thermal solvent/non-solvent, supercritical/subcritical fluid technology and microsphere coupling, and fiber bonding, among others [1,2,3,4,5,6,7,8,9,10,11,12]. Some of these processes are highly advantageous in fabricating 3D scaffolds, such as supercritical/subcritical fluid sintering, as these processes do not utilize any toxic or harmful substance and are carried out in relatively mild conditions, causing no loss of growth factors or drugs [2,6,12]. In this framework, Sadeghi et al. [13] fabricated composite scaffolds of polyhydroxybutyrate (PHB) and chitosan by using the electrospinning process. They demonstrated that the composite material for cartilage repair could be quickly degraded with the addition of chitosan. However, the adhesion of chondrocytes on the surface of the scaffold was significantly high, yielding biomimetic architectures. In another study, Carfì and coworkers [14] used poly-l-lactic acid- polyhydroxyethylaspartamide-polylactic acid (PLLA-PHEA-PLA) monomers to fabricate porous scaffolds by thermal phase separation technology. The biocompatible, as well as the hydrophilic PLLA-PHEA-PLA porous scaffolds, significantly augmented the chondrocyte adhesion and proliferation. Moreover, various 3D scaffolds based on poly(lactide-*co*-glycolide)/poly(ε-caprolactone) (PLGA/PCL) microspheres were fabricated for osteochondral repair [15]. Despite the advantageous properties and successful engineering of chondrocytes, the applicability of these approaches is limited due to certain limitations such as the residue of pore-forming agents and organic solvents, the difficulty in controlling the shape of the pore, the complexity of the preparation process, which is expensive and requires extreme conditions such as high temperatures and shear stress, among others.

To this end, 3D printing, a rapid prototyping technology based on the principle of material dispersion or accumulation, was developed to generate geometrically defined porous architectures in 3D [7,8,9,10]. This biocompatible strategy efficiently fabricates biomimetic tissues that are physiological relevant by overcoming certain limitations associated with the conventional approaches intended for scaffold generation, such as the utilization of porogens and organic solvents, poor control over the fabrication of pores, moderate porosity, poor reproducibility, and challenges in scale-up, among others. Several reports have demonstrated the printing of 3D porous scaffolds such as gelatin (Gel) [16], nano-hydroxyapatite (n-HA) [17,18], and PLGA [19,20]. However, the combination of polymers in 3D printing is advantageous as they suffer from specific problems when used individually, such as poor mechanical properties, poor hydrophilicity, and low cell adhesion rate, among others.

In recent times, considerable efforts have been dedicated to developing 3D architectures, focusing on the effect of growth factors on cell growth and differentiation using various technologies [21,22]. However, most of these studies focused on the utilization of growth factors for osteoblast differentiation [23,24,25,26]. On the other hand, the utilization of growth factors suffers from specific challenges which limit their applicability, such as a complicated extraction process, high cost, and reduced yields. Moreover, this method has potential toxicity, carcinogenicity, and ectopic osteogenesis [24,25,26,27,28,29,30]. There is a critical need to explore a simple, effective, safe, and inexpensive bioactive substance that can replace growth factors as an actual requirement for cartilage repair. Icariin (ICA) has attracted increasing attention from researchers because it is cheap, easy to obtain, and promotes osteochondral defect repair [31].

**Figure 1:**
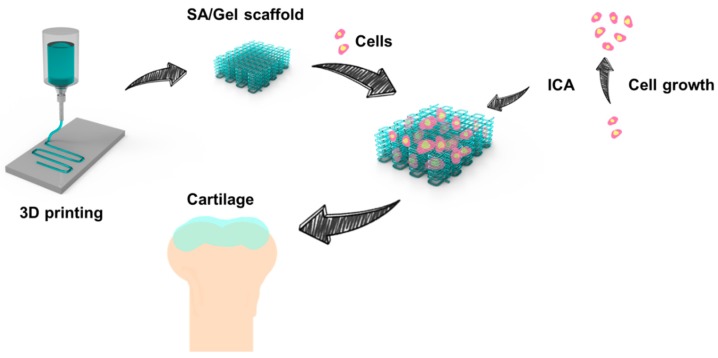
Schematic representation showing the procedure of fabricating the sodium alginate (SA)/gelatin (Gel) scaffolds using the three-dimensional (3D) printing strategy in sequential steps. The procedure includes the printing of 3D scaffolds using a composite of polymer inks into a microfibrous bed, seeding of chondrocytes into the scaffold bed, and the addition of icariin (ICA) for their differentiation.

Inspired by these facts and the problems associated with osteochondral defects, we fabricated cytocompatible, biomimetic, and porous 3D scaffolds based on 3D printing technology for cartilage repair (Figure 1). In a typical 3D printing process, the highly cytocompatible and biodegradable materials of sodium alginate (SA) and Gel were used to print the 3D scaffolds with highly porous architectures and excellent mechanical strength. Various physicochemical attributes such as thermal stability, swelling rate, cell distribution, proliferation, and chondrogenicity within the scaffolds were explored to prove the efficiency of the 3D-printed scaffolds. Furthermore, the cartilage repair was explored with ICA treatment after exposure to 3D-printed scaffolds.

## 2. Materials and Methods

### 2.1. Materials

SA and Gel were purchased from Daigang Biological Co., Ltd. (Jinan, China). Absolute ethanol (99.8% purity), glutaraldehyde (99.8% purity), dual acridine orange/ethidium bromide staining (AO-EB staining) calcium chloride (CaCl_2_), 3-(4,5-dimethylthiazol-2-yl)-2,5-diphenyltetrazolium bromide (MTT), 1,9-dimethyl methylene blue (DMMB), chondroitin sulfate, glutaraldehyde (99.8% purity), and dichloromethane (DCM, 99.8% purity) were obtained from Sigma Co., Ltd., St. Louis, MO, USA, Penicillin, Dulbecco’s modified Eagle’s medium (DMEM), fetal bovine serum (FBS), and streptomycin were obtained from Gibco Co. Ltd. All other compounds were of analytical purity and used without any further purification.

### 2.2. Fabrication of SA/Gel Scaffolds

Initially, the colloidal solutions were pretreated by dissolving 2 g of SA and 6 g of Gel in 50 mL of dd-H_2_O. This colloidal solution was used to print the 3D microfibrous scaffolds based on the extrusion-based printing using a commercial 3D printer (Regenovo 3D Bio-Architect, Hangzhou, China). In this study, we critically optimized the conditions for printing by choosing the following conditions (printing pressure of 0.20–0.40 MPa, line spacing of 0.3–1.2 mm, printing diameter of 210–510 µm, and printing speed of 4–16 mm/s). However, the printing temperature (200 °C) was kept constant throughout the printing process. The printed SA/Gel composite scaffold was quickly soaked in 5% CaCl_2_ for 30 min for rapid solidifying, and then soaked in 2% glutaraldehyde for 24 h to complete the crosslinking. The residual glutaraldehyde was removed with L-glutamic acid treatment, and finally the scaffolds were freeze-dried and subjected to further studies.

### 2.3. Physical Characterizations of the Composite Scaffolds

The thermal stability of the composite scaffolds was determined by thermogravimetric analysis (TGA). The samples were dried, and thermal analysis was performed by increasing the temperature from ambient to 800 °C at a heating rate of 20 °C/min under strict nitrogen supply. The surface morphology of the scaffolds was elucidated using a field emission scanning electron microscope (FE-SEM, S-4800, HITACHI, Tokyo, Japan). The samples were prepared by being adhered onto an aluminum stub with a thin self-adhesive carbon film and then coated with an ultrathin layer of gold, and the images were captured. A confocal laser scanning microscope (CLSM, TCS SP8, Leica, Wetzlar, Germany) was used to capture the stained cells. The swelling rate of the samples was determined by placing them in buffers mimicking physiological fluids. The accurately weighed SA/Gel-based 3D scaffolds after drying (W_0_) were placed in a tube containing phosphate buffered saline buffer (PBS, 5 mL, pH = 7.4) for 1 h. The scaffolds were wiped with filter paper to remove the water adsorbed, and the mass of the scaffolds was recorded (W_x_). Finally, the swelling rate was calculated using following the formula:

W% = (W_x_ − W_0_)/W_0_ × 100 (1)

The degradation rate of the 3D scaffolds was determined by following a procedure similar to that used to determine the swelling rate. However, the difference in the weights of the scaffolds in the tubes that placed in an orbital shaker (37 °C, 65 rpm) was recorded at predetermined time intervals. Finally, the degradation rate was calculated by using the following formula:

M% = (M_x_ − M_0_)/M_0_ × 100 (2)

where M_0_ and M_x_ represent the weight of the sample before and after treatment, respectively.

### 2.4. Cytocompatibility Study

For the cell culture, C5.18 cells were obtained from the American Type Culture Collection (ATCC, Manassas, VA, USA). The cells were cultured in DMEM supplemented with 20% FBS, 100 U mL^−1^ of penicillin, and 100 µg mL^−1^ of streptomycin. The cells were incubated in a humidified atmosphere maintained at 37 °C and 5% CO_2_.

We investigated the cytocompatibility of these polymeric 3D scaffolds by MTT assay. Initially, the experimental scaffolds were sterilized and then activated by soaking in 75% of ethanol for 30 min. Subsequently, they were washed thrice with PBS. The C5.18 cells were seeded at a density of 5 × 10^3^ cells/well of a 96-well plate for proper cell attachment. After 24 h, the media was removed and replaced with the scaffold leaching solution (experimental groups) along with the media alone (negative control), or with DMSO (positive control, 5% in media). After 4 h, media containing FBS were added to supply the nutrients. Under the similar conditions provided at the predetermined intervals (1, 2, and 3 days) of incubation, 10 µL of MTT solution was added to each well followed by incubation for a further 4 h. The content in the well was discarded, and 100 µL of DMSO was added before a further incubation for 10 min to dissolve the formazan crystals crystal completely. Finally, the absorbance was measured using the microplate reader at a wavelength of 570 nm.

### 2.5. Effect of ICA on Chondrocyte Growth

#### 2.5.1. Chondrocyte Proliferation

The proliferation of C5.18 cells in the presence of ICA was measured using the MTT assay. Cells were seeded at a density of 2 × 10^3^ in each well of a 96-well plate and incubated for 24 h for proper cell attachment. The media was then removed and replaced with the media containing different concentrations of ICA. After incubation for 1, 3, 5, and 7 days, MTT solution (20 µL) was added and further incubated for 4 h. Later, the procedure was continued as mentioned above (see cytocompatibility assay).

#### 2.5.2. Determination of Glycosaminoglycan (GAG) by DMMB Assay

The secretion of glycosaminoglycan (GAG) from chondrocytes was measured by the DMMB method. The principle behind the operation is that the compound DMMB binds explicitly to GAG, resulting in a change of color from blue to pink. The extent of color variation can be determined, and the GAG can be quantified by measuring the absorbance at 595 nm against the standard curve with chondroitin sulfate (Sigma). The cells were seeded at a density of 3 × 10^4^ per well of a 24-well plate and then incubated for 24 h for proper cell attachment. Later, different concentrations of ICA were added and cultured for 7 and 14 days along with the control group (no treatment). Then, the cell culture media (150 µL) was extracted, and 3 mL of DMMB chromogenic reagent was added and incubated for 15 s. The optical density (O.D.) was then determined at 525 nm. The absorbance value was calculated according to the standard curve of chondroitin sulfate, and the GAG content of chondrocytes was calculated.

### 2.6. Chondrocyte Differentiation on SA/Gel Scaffolds

#### 2.6.1. Cell Adhesion and Distribution

A cell adhesion experiment using chondrocytes on the designed 3D scaffolds was performed according to following the procedure. Briefly, the 3D scaffolds were pretreated for activation by placing them in PBS for 2 h. Later, the cells (1 × 10^6^ per mL) were placed into the wells containing scaffolds. After incubation for predetermined time intervals (1, 3, 5, and 7 h), the cells were fixed with 2.5% glutaraldehyde solution for 24 h and washed thrice with PBS solution to remove the non-adherent cells. Later, the adherent cells on the scaffolds were observed using SEM imaging.

The distribution of chondrocytes on the scaffold surface was observed by AO staining, as it can enter through the cell membrane and bind with the nuclei, resulting in the emission of bright green fluorescence under laser excitation. First, the cells cultured over the surface of the scaffold were separated and then stained with a configured AO for 3 min. Later, the composites were washed thrice with PBS to remove the excess dye and then observed using CLSM imaging at a wavelength of (λ_EX_) 488 nm. Further, the experiments were repeated for determining the dead cells using dual stain AO-EB staining under CLSM at excitation wavelengths of 488 and 536 nm, respectively.

#### 2.6.2. Chondrocyte Proliferation

The SA/Gel scaffolds used in the experiment were activated by soaking in 75% ethanol for 30 min. They were then washed with PBS to remove the residues of ethanol. The cells were seeded onto the SA/Gel scaffolds at a density of 1 × 10^6^ per mL in a 48-well plate. After 24 h, the media was replaced with different concentrations of ICA and incubated for 3, 5, and 7 days. Then, the MTT solution was added and the sample incubated for 4 h. Finally, the media was discarded, and the remaining procedure of the MTT assay was continued.

#### 2.6.3. GAG Secretion

The C5.18 cells were seeded onto 24-well plates containing the SA/Gel scaffolds. Further, the scaffolds were exchanged with media containing different concentrations of leaching solutions of ICA. After 7, 14, and 21 days of incubation, 1 mL of papain solution was added and maintained at room temperature for 24 h for freeze-drying. For GAG measurement, each sample was dissolved in 1 mL of PBS, then 150 µL was taken after centrifugation and filtration, 3 mL of DMMB solution was added for 15 s, and the absorbance was detected at a wavelength of 525 nm. According to the standard curve of chondroitin sulfate, the content of GAG was calibrated.

### 2.7. Statistical Analysis

All results subjected to significance tests are presented as the mean ± standard deviation (*n* = 3). The statistical analysis of all experimental data was performed using SPSS version 19.0. Analysis of variance (ANOVA) single factor analysis was conducted at a defined level of statistical significance, specifically *p* < 0.05.

## 3. Results and Discussions

### 3.1. Optimization of Processing Parameters

In this study, we demonstrate the fabrication of polymeric scaffolds with excellent mechanical strength based on a combination of polymers, i.e., SA and Gel, using 3D printing technology for cartilage repair. Initially, various appropriate conditions feasible for the printing of 3D scaffolds such as printing temperature as well as speed and needle diameter were systematically optimized. Then, the scaffolds printed at the optimized conditions were systematically characterized using various physical characterization techniques. Later, the cytocompatibility of these scaffolds was measured using the MTT assay. To overcome the limitations of growth factors in the cell differentiation process, we herein tested the efficacy of ICA in the presence of scaffolds for efficient chondrocyte differentiation. Prior to evaluation, we demonstrated the effects of ICA on chondrocyte growth. Finally, cell proliferation, adhesion, and growth were demonstrated by various techniques such as SEM and CLSM investigations, and the chondrogenic differentiation was demonstrated by the determination of GAG levels.

**Figure 2:**
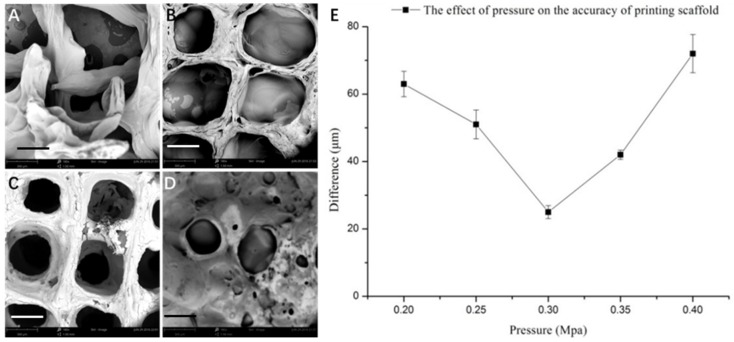
Effect of printing pressure on the morphology of scaffolds. (**A**-**D**) SEM images of scaffolds obtained by 3D printing at different pressures: (**A**) 0.20, (**B**) 0.25, (**C**) 0.30, and (**D**) 0.40 MPa (scale bar: 300 μm). (**E**) Graphical representation showing the accuracy of printing the scaffolds at different printing pressures.

**Figure 3:**
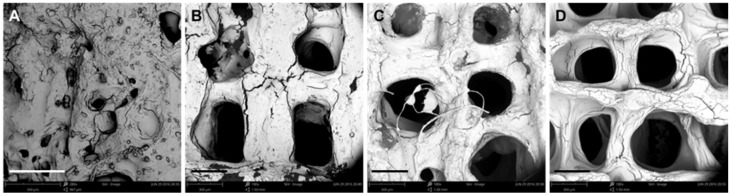
Effect of printing speed on the morphology of scaffolds. (**A**–**D**) SEM images of scaffolds obtained by 3D printing at different speeds: (**A**) 4, (**B**) 8, (**C**) 12, and (**D**) 16 mm/s (scale bar: 300 μm).

It is evident that the printing parameters had a significant effect on the morphology of the 3D SA/Gel scaffolds, which may eventually affect the cell adhesion, growth, and differentiation. Briefly, at lower printing pressures, the scaffolds were broken, resulting in irregular shapes (Figure 2). With the increase of pressure to 0.3 MPa, the morphology was intact, and the pores were well lined. Further, the increase of pressure to 0.4 MPa resulted in the complete collapse of the support with no more extended 3D arrangement, revealing that only the optimized printing pressure of 0.3 MPa resulted in 3D architectures. On the other hand, the variation in the printing speed also had a significant influence on the accuracy of printing the scaffolds. The results obtained at different speeds with the optimum printing pressure of 0.3 MPa demonstrated that the 3D orientation of structures gradually increased, with 16 mm/s being the optimum speed for printing (Figure 3). Contrarily, the slower printing speed resulted in a much more substantial amount of thread per unit time.

**Figure 4:**
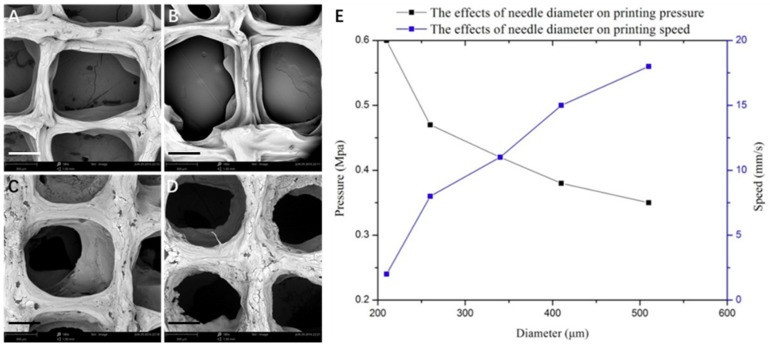
Effect of needle diameter on the morphology of scaffolds. (**A**–**D**) SEM images of scaffolds obtained by 3D printing at different needle diameters: (**A**) 210, (**B**) 260, (**C**) 410, and (**D**) 510 µm (scale bar: 300 μm). (**E**) Graphical representation showing the effect of needle diameter on printing pressure and printing speed.

**Figure 5:**
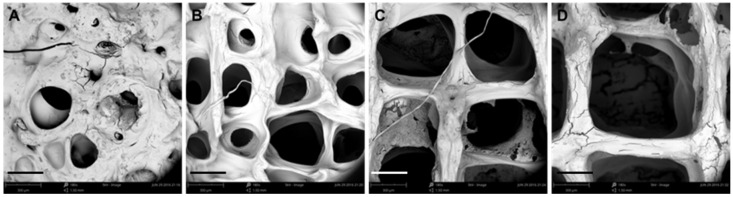
Effect of printing line spaces on the morphology of scaffolds. (**A**–**D**) SEM images of scaffolds obtained by 3D printing at line spaces: (**A**) 0.3, (**B**) 0.6, (**C**) 0.9, and (**D**) 1.2 mm (scale bar: 300 μm).

In addition to printing speed and pressure, the diameter of the nozzle also played a crucial role in the printing of 3D architectures (Figure 4). From the scaffold morphology, the regularity of the scaffolds and the porosity increased significantly with the gradual increase of the diameter of the scaffold lines. The results demonstrated that the increase of the needle diameter improved the accuracy of the scaffold (Figure 4A–D). Considering the swelling characteristics of the scaffolds as well as the nominal size of the cell, it is suitable to use the medium size printing needle to prepare biomimetic 3D scaffolds. Figure 4E shows the effect of increasing the diameter of the printing needle on the pressure and printing speed required for the preparation of the scaffold. The experimental results showed that, with the increase of the needle diameter, the printing pressure was reduced and the printing speed was augmented. The decrease of printing pressure is beneficial to reduce the damage of the biological printing process to the cell activity in the scaffolds, and the increased printing speed would improve the efficiency of the tissue scaffold preparation. With the enlargement of the diameter of the printing needle, the porosity of the scaffold increased gradually. Considering the diameter of tissue cells from more than 10 microns to dozens of microns, the TE scaffolds prepared at around a 300-µm print diameter can meet the requirements for the growth and colonization of chondrocytes. Another parameter that played a crucial role in the printing of 3D architectures for biomedical applications is the printing spacing, which significantly affects the shape and accuracy of the scaffolds (Figure 5). The experimental data demonstrated that the spacings of 0.3 and 0.6 mm had a significant influence on the shape of the scaffold, resulting in irregular architectures with no clear pores due to the close interline adhesion and intertwining of the lines at the close gel lining. At the outset, the optimized parameters were used further to fabricate 3D scaffolds, and they were then systematically characterized using various techniques.

### 3.2. Physical Characterizations

**Figure 6:**
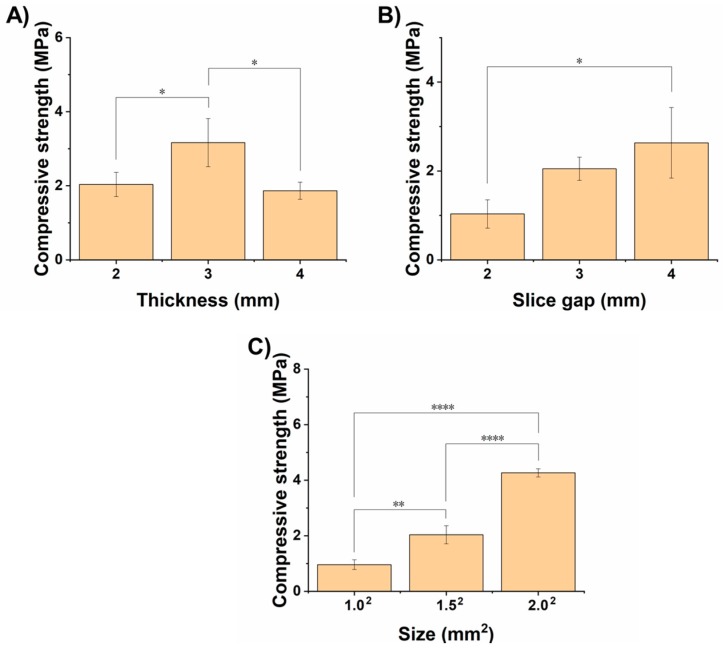
Mechanical properties of scaffolds. Graphical representation showing the changes in the compressive strength, indicating the mechanical properties of SA/Gel at different (**A**) thicknesses, (**B**) slice gaps, and (**C**) overall sizes of the scaffolds. * represents *p* < 0.05, ** represents *p* < 0.01, **** represents *p* < 0.0001.

After optimizing various printing parameters, we printed SA/Gel scaffolds at the optimized printing conditions of various dimensions and their effects on the mechanical properties, i.e., the compressive strength of the scaffolds, was investigated. Herein, the SA/Gel scaffolds with different thicknesses, slice gaps, and final sizes of the scaffold constructs were prepared, and their compressive strength was measured. As shown in Figure 6A, the compressive strength of the scaffolds increased with the thickness from 2 to 3 mm, but surprisingly it reduced at 4 mm, indicating that a thickness of 3 mm is optimum. Further, the slice gap had a significant influence on the compressive strength, which increased with the increase in the gap between the slices. Similarly, the strength of the scaffold also followed the effect of the slice gap, demonstrating that the overall size of the scaffold also had a significant influence on the compressive strength. It is evident from Figure 6C that the increase in the size of the final scaffold architecture enhanced the compressive strength by 2-fold from 1 to 1.5 mm^2^ and then subsequently to 2 mm^2^. Together, it is evident that these results represent that SA/Gel scaffolds exhibit better mechanical properties suitable for TE.

**Figure 7:**
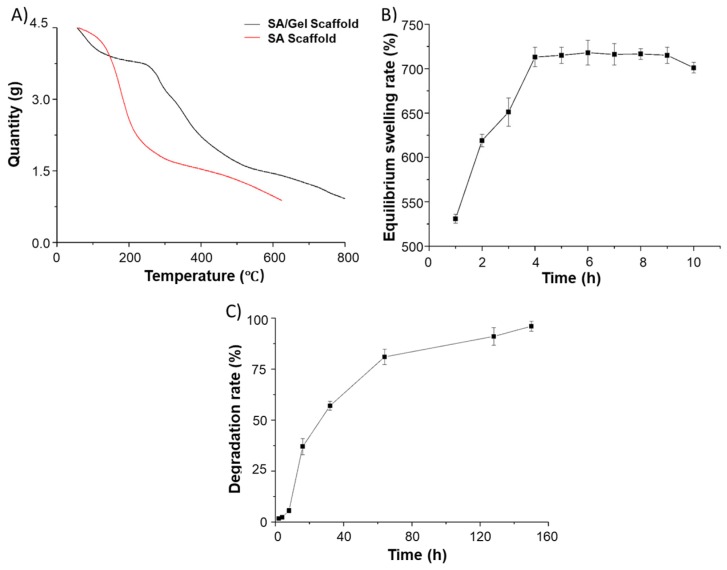
Physical characterization of SA/Gel 3D porous scaffolds. (**A**) Thermogravimetric analysis (TGA) curves of composite (SA/Gel) as well as SA scaffolds. (**B**) Equilibrium swelling rate and (**C**) degradation rate of the composite porous SA/Gel scaffolds.

Initially, the weight loss events were determined by TGA analysis to confirm the presence of different polymers (SA and Gel) in the 3D scaffolds by scanning against the SA scaffolds. As shown in Figure 7A, the TG curves show that a significant weight loss event ranging from 180 to 600 °C represents the carbon residues of SA/Gel scaffolds, which were higher than those of the weight loss in scaffolds printed with SA alone. It is evident from the TGA curves that there exist specific interactions between SA and Gel due to their thermal stability. From simple click chemistry approaches, it is considered that the OH- and COO- groups of alginate polymer interact with the NH_2_ and COO- groups of Gel, resulting in the formation of hydrogen bonds and electrostatic interactions. This finding demonstrates the interpenetration of the alginate as well as Gel molecules, reached at the molecular level, thus facilitating the compatibility of these polymers.

Furthermore, the swelling rate of the composite scaffolds was observed in order to explore its efficacy in biomedical applications. Often, compounds with higher hydrophilicity possess enormous water absorption capacities that facilitate the wettability of the material and subsequently enable the adhesion, proliferation, and migration of cells during the differentiation process. Figure 7B depicts that the maximum swelling rate of the composite scaffold reached 713% in 4 h of incubation. Moreover, the size of the scaffold increased close to 62% after cross-linking and freeze-drying, which should be attributed to the strong hydrophilic and the swelling ability of SA and Gel polymers. Interestingly, in a short time, the dry gel was rapidly swelled into the hydrogel, demonstrating the beneficial property of composite scaffolds toward the adhesion and growth of chondrocytes.

The degradation of biomimetic architectures in the body is an essential physicochemical attribute to be considered during the fabrication of biomedical devices, especially the biomedical stent/scaffolds intended for TE. Moreover, it should be noted that synthetic materials often result in severe consequences of immune reactions, leading to organ damage and, in a few instances, death. A slower degradation rate is commonly anticipated during TE, as the regeneration process is slow, being is predominantly considered for bone TE. Thus, this rate critically depends on the type of cells and their growth rate. To explore this attribute, we investigated the degradation ability of SA/Gel composite 3D scaffolds in buffer mimicking physiological fluids. Figure 7C depicts the degradation behavior of the composite scaffolds that were subjected to buffers in physiological fluids at a constant temperature. It was found that the scaffolds showed a minimal amount of degradation in the first 10 h, but the degradation ability subsequently enhanced and the scaffolds were degraded entirely in a week. The plausible reasons behind this degradation might be the extensive porosity, with an optimum pore size of 200~300 µm, and the liquid surface tension that should be overcome when the support comes in contact with the physiological fluids. Moreover, the crosslinking of the scaffolds with CaCl_2_ happened to be favorable on the surface of the scaffolds, which remained intact for a specified period and on further incubation resulted in the complete degradation of the scaffolds. Although the in vitro degradation is rapid, the essential considerations such as cytocompatibility and the growth rate of cells in the presence of growth regulators yielding extracellular matrix (ECM) play a significant role in tissue regeneration. However, it should be noted that, in some instances, the cell-produced ECM matrix may delay the degradation.

### 3.3. Cytocompatibility

**Figure 8:**
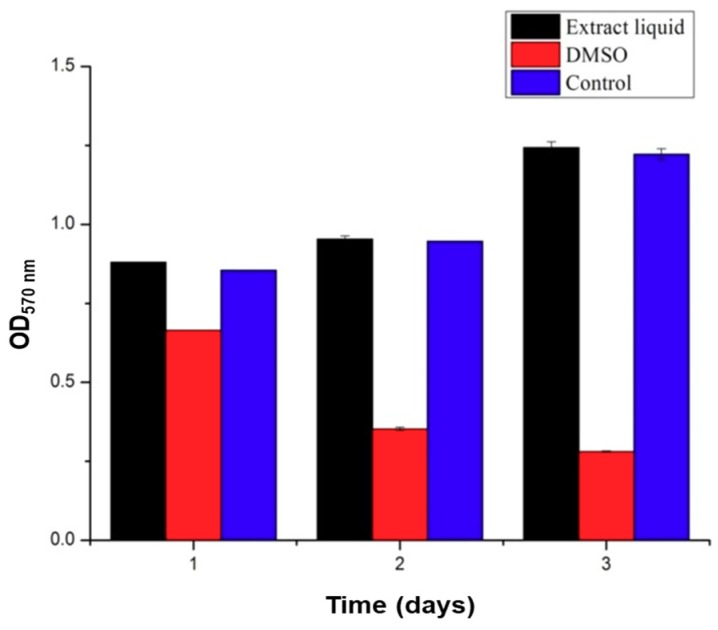
Cytocompatibility assessment of scaffolds on chondrocytes. MTT assay results showing the viable number of cells after treatment with the leaching solutions of scaffolds (10 mg/mL) along with negative (media alone) and positive controls (DMSO).

The polymers used in this study, SA and Gel, are generally considered biocompatible materials, which are extensively utilized in biomedical applications including drug delivery and regenerative medicine. Though the materials are biocompatible, the compatibility of the medical devices, in some instances, depends on the processing steps followed and the solvents utilized during the fabrication, which may lead to severe consequences of incompatibility. In this context, previous reports indicated that the presence of glutaraldehyde showed certain signs of cytotoxicity [32]. Thus, it is a prerequisite to measure the biocompatibility attribute in that application area. The cytotoxicity test of the scaffolds was performed using C5.18 cells at different exposure times of 1, 2, and 3 days using the MTT assay. The results are illustrated in Figure 8. The results demonstrated that the scaffolds had no significant influence on the viability of cells and there existed no significant difference between the experimental group and the negative control group. Contrariwise, the addition of 5% DMSO, denoted as positive control group, showed significant inhibition on the growth of chondrocytes. It is evident from the results that the addition of glutaraldehyde during the preparation of scaffolds to crosslink the Gel and strengthen the scaffolds had no effect on viability, revealing that the excess glutaraldehyde was removed entirely by l-glutamic acid. Moreover, the operating conditions of 3D printing and processing the scaffolds are highly cytocompatible, indicating that no significant changes in the functional groups were observed.

### 3.4. Effect of ICA on Chondrocyte Growth

**Figure 9:**
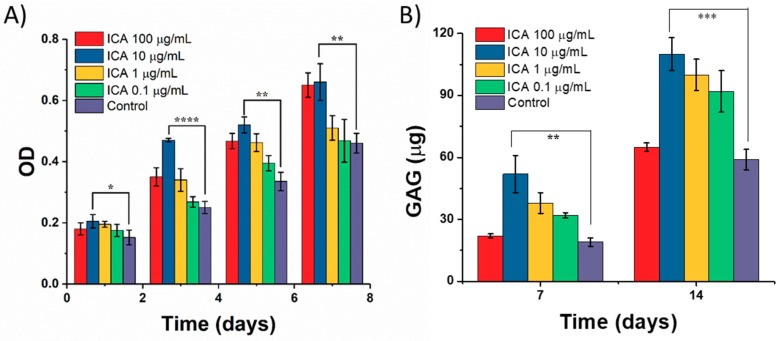
Effect of ICA on chondrocyte culture. The effect of ICA on (**A**) the proliferation of chondrocytes. (**B**) The secretion of glycosaminoglycan (GAG) (µg) from chondrocytes. (* represents *p* < 0.05, ** represents *p* < 0.01, *** represents *p* < 0.005, and **** represents *p* < 0.0001).

Initially, the effect of ICA on chondrocyte growth was determined by measuring the proliferation of C5.18 cells using an MTT assay after incubation for 1, 3, 5, and 7 days. As shown in Figure 9A, it is evident that there was no significant effect of ICA on cell proliferation compared with that of the control group after ICA treatment in the beginning (Day 1). With the increase of culture time, the proliferation of cells varied significantly, with different concentrations of ICA ranging from 0.1–100 µg/mL. Through the analysis and comparisons, we concluded that ICA had a significant cell proliferation effect on the growth of cartilage cells with increasing the ICA concentration. However, a slight inhibition of the proliferation of chondrocytes was observed at 100 µg/mL. Still, the experimental results at low concentration of ICA promoting cell proliferation and a certain degree of inhibition at high concentrations were consistent with the conclusions of Yang et al. [33].

Further, we extended our studies toward the differentiation of chondrocytes by determining the GAG secretion in exploring the effect of ICA on chondrocyte culture. The activity and functional state of C5.18 cells that differentiated into chondrocytes can be determined by detecting the content of GAG secreted by cells in the scaffolds, which is the primary and most acceptable marker for chondrocyte differentiation [34,35]. GAG is a major and important component of the cartilage ECM, which is formed by covalent bonding between chondroitin and keratin sulfate [36]. GAG plays a major role in providing the biological signals to stem cells and chondrocytes for the development and structural as well as functional restoration of cartilage. In particular, sulfated GAGs can bind to growth factors and enhance their functionality through interactions [36]. Therefore, the measurement of the GAG content is highly appropriate in determining chondrocyte proliferation and cartilage ECM formation. Figure 9B depicts the content of GAG secreted by C5.18 cells after exposure to different concentrations of ICA at different incubation time intervals. The results showed that the GAG content of chondrocytes increased gradually with the prolongation of culture time from 7 to 14 days of incubation. Overall, the ICA at a concentration of 10 µg/mL exhibited a very significant promoting effect. However, the secretion ability of chondrocytes at 100 µg/mL of ICA treatment group was reduced compared to that of 10 µg/mL of ICA. Together, the data represented that 10 µg/mL of ICA concentration can significantly promote the secretion of GAG in chondrocytes.

### 3.5. Chondrocyte Growth and Differentiation on 3D-Printed Scaffolds

**Figure 10:**
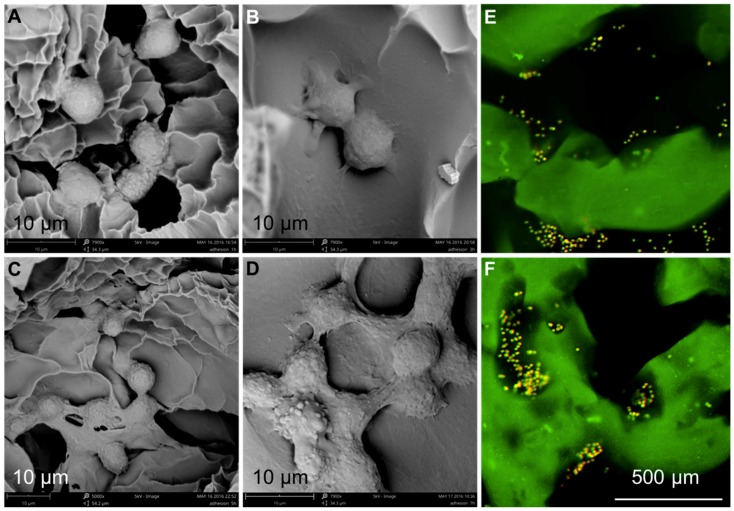
Chondrocytes distribution on the designed 3D-printed scaffolds. (**A**-**D**) SEM images showing the adhesion of chondrocytes after (**A**) 1, (**B**) 3, (**C**) 5, and (**D**) 7 h (scale bar—10 μm, magnifications: 5000× for panel c, and 7900× for panels a, b, and d). (**E**,**F**) Confocal laser scanning microscope (CLSM) images showing the distribution of cells over the scaffolds at different magnifications.

The adhesion of chondrocytes was investigated using various techniques. Initially, the morphological features of the scaffolds incubated with chondrocytes were observed and then confirmed by fluorescence imaging. As shown in Figure 10A–D, the SEM images of SA/Gel scaffolds seeded with chondrocytes for different periods showed that the cells were attached to the surface of the scaffold after 1 h. However, the morphology of the cells did not stretch and still remained in the spherical state, similar to their state of suspension. With the increase of incubation time to 3 h, the cell morphology was gradually extended, resulting in the appearance of “pseudopodia” with significant contact to the surface of the scaffolds. The adhesion was significantly augmented after 5 h, spreading entirely on the surface and showing the irregular polygon shape of the cells. At 7 h, the cells began to interact with each other and were aggregated to form cell cysts due to the proliferation of cells on the surface of the scaffold. Furthermore, the adhesion and distribution of cells were confirmed by CLSM imaging. After the chondrocytes were inoculated onto SA/Gel scaffolds, the cell-scaffold composite was stained with AO dye after 1 h, and the distribution of the cells on the scaffold was observed at an excitation wavelength of 488 nm. Figure 10E,F show the distribution of cells on scaffolds after the inoculation of chondrocytes, demonstrating that most of the cells inoculated onto the scaffolds were distributed in the folds and pores of the scaffolds, and only a few were distributed on the surface of the scaffolds. These results also indicate the importance of adhesion sites on the scaffolds, augmenting the distribution and adhesion of chondrocytes.

**Figure 11:**
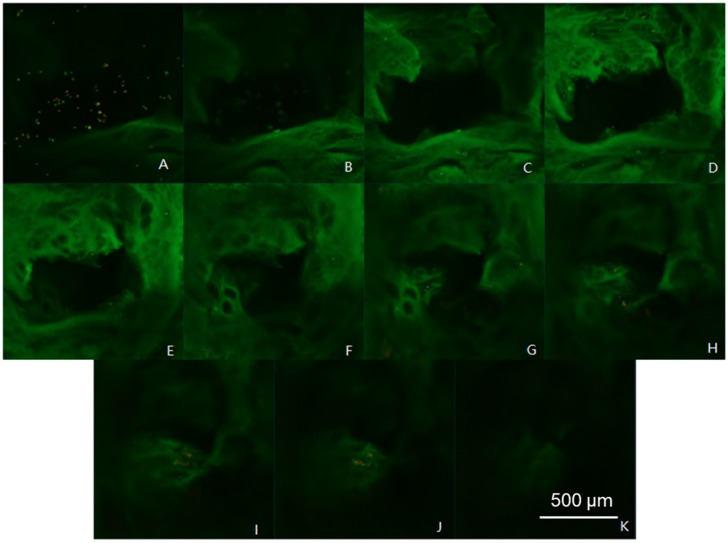
Chondrocyte growth over the scaffolds at different layer thicknesses by 3D scan fluorescence imaging on the z-axis at a thickness of 50 μm. (**A**–**K**) 0–550 μm.

The viability, as well as the arrangement of cells in different layers of the scaffold, is shown in Figure 11. The experimental results showed that a large number of cells were deposited on the surface of the scaffold, and the number of cells increased with the thickness of the scaffolds. On the other hand, after 3 days, the cells showed a certain degree of death or apoptosis. This showed that the structure of the scaffold is beneficial for the survival of the cells, and the marginal survival rate of the cells in the gel scaffold is high, i.e., the phenomenon of “marginalization”.

**Figure 12:**
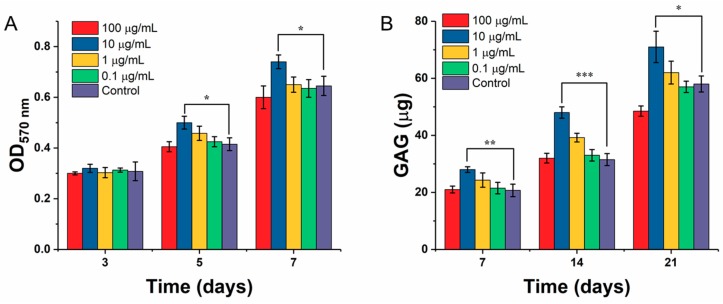
Chondrocyte growth and proliferation over the designed SA-Gel scaffolds in the presence of ICA. (**A**) Cell proliferation by MTT assay; (**B**) GAG secretion by DMMB assay on the SA-Gel scaffolds in the presence of ICA. (* represents *p* < 0.05, ** represents *p* < 0.01, and *** represents *p* < 0.005).

The effect of gradient ICA on the proliferation of chondrocytes inoculated on the scaffolds is shown in Figure 12A. The experimental results showed that the ICA concentration of 10 µg/mL in the experimental group was significantly higher than that of the control group (*p* < 0.05), but there was no significant difference between the other gradient groups and the control group. After culturing for 5 and 7 days, 10 µg/mL of ICA showed a significant difference in promoting the proliferation of chondrocytes on scaffolds compared with that of the control group (*p* < 0.01). It was found that the absorbance of the ICA concentration of 100 µg/mL at 5 days was slightly lower than that of the control group. Compared with the control group, the proliferation of the cells on the scaffold was inhibited after 7 days in the 100 µg/mL of ICA-treated group, and there existed a significant difference. Together with the increase of culture time, ICA showed a dose-dependent effect on the proliferation of chondrocytes. Furthermore, the differentiation of chondrocytes on the 3D-printed scaffolds was measured by GAG secretion. Figure 12B depicts the content of GAG secreted by C5.18 cells at different concentrations of ICA. The results demonstrated that the GAG content of chondrocytes increased gradually with the prolongation of culture time, demonstrating that the GAG secretion is time-dependent as well as dose-dependent. However, the dose of 10 µg/mL had a higher secretion effect, and a further increase in dose reduced the secretion.

## 4. Conclusions

In this study, we fabricated microfibrous polymeric scaffolds using a 3D printing technique for cartilage repair. The composite SA/Gel scaffolds offered better thermal stability, swelling ability, and degradation rate, which could be beneficial for regenerative medicine. The porous architectures facilitated enough room for the adhesion and growth of chondrocytes. Interestingly, the ICA addition further promoted the proliferation of cells and conveniently supported their differentiation. This approach may further endorse the integration of multiple cell types, as well as multiple growth enhancers such as ICA, in the presence of the 3D porous scaffolds to engineer biomimetic tissues.
